# Retinal Changes After Posterior Cerebral Artery Infarctions Display Different Patterns of the Nasal und Temporal Sector in a Case Series

**DOI:** 10.3389/fneur.2020.00508

**Published:** 2020-06-05

**Authors:** John-Ih Lee, Laura Boerker, Lena Gemerzki, Jens Harmel, Rainer Guthoff, Orhan Aktas, Michael Gliem, Sebastian Jander, Hans-Peter Hartung, Philipp Albrecht

**Affiliations:** ^1^Department of Neurology, Medical Faculty, Heinrich-Heine-University, Duesseldorf, Germany; ^2^Department of Ophthalmology, Medical Faculty, Heinrich-Heine-University, Duesseldorf, Germany

**Keywords:** retinal layer, optical coherence tomography, multifocal visual evoked potential, posterior cerebral artery infarction, ischemic stroke

## Abstract

**Background:** Visual field defects are a common and disabling consequence of stroke and a negative prognostic factor of patient's quality of life. They result from lesions in different parts of the visual system, most commonly the visual cortex and optic radiation. An important pathophysiological mechanism is transsynaptic retrograde degeneration (TRD).

**Methods:** In a case series 21 patients with posterior cerebral artery (PCA) territory infarctions were analyzed by spectral-domain optical coherence tomography (SD-OCT) and multifocal visual evoked potentials (mfVEPs) cross-sectionally and longitudinally for up to 6 months. In OCT, symptomatic affected nasal and temporal sectors and corresponding visual fields in mfVEPs were compared to the contralateral side.

**Results:** SD-OCT revealed a significant reduction (−2.92 ±2.53 μm, mean ± SD) of the symptomatic nasal macular retinal nerve fiber layer (RNFL) thickness and of the symptomatic temporal peripapillary RNFL after 6 months compared to baseline whereas the symptomatic temporal macular quadrant already showed a significantly thinner RNFL at baseline. The mfVEP first peak latency at baseline was significantly different (nasal visual field +11.69 ±11.17 ms, mean ± SD; temporal visual field +16.63 ±7.97 ms, mean ± SD) on the symptomatic compared to the asymptomatic field. The nasal visual fields partly recovered in amplitude and first peak latency of mfVEPs over the following 6 months compared to baseline.

**Conclusion:** The dynamics of OCT and mfVEP outcomes for degeneration and recovery after PCA infarction differ between the nasal and temporal retinal sector. We postulate that retinal sectors may differ in their temporal pattern of TRD over time after retrogeniculate cerebral infarction.

## Introduction

Visual field defects (VFDs) affect approximately one half of acute stroke patients and about 25% of patients in the chronic phase post-stroke (1, 2). Despite having an enormous impact on patients' autonomy and quality of life (3, 4), thorough diagnostic evaluation and specific visual rehabilitation are rare (5). VFDs are caused by damage to the visual pathway, most commonly by stroke affecting the visual cortex in the posterior cerebral artery (PCA) territory or the optic radiations. Due to its linearity and accessibility to structural and functional examinations the visual system is particularly suitable to study neurodegeneration. Elucidating neurodegenerative mechanisms after stroke and identifying meaningful and non-invasive markers would facilitate clinical trials on rehabilitative, neuroprotective and neuroregenerative strategies. Previous case series and small trials have indicated that, in addition to the Wallerian degeneration of damaged neurons, there are transsynaptic retrograde neurodegenerative processes that might worsen or solidify visual disability in the long-term (6–15). Spontaneous recovery by functional reorganization and the effectivity of existing treatment strategies such as behavioral therapies or brain stimulation are limited. Thus, new treatment strategies are warranted. However, the underlying pathophysiological processes including retrograde transsynaptic neurodegeneration in humans are not well understood. To date only a few relevant studies examining the dynamics of these processes longitudinally have been conducted (16). Optical coherence tomography (OCT) allows noninvasive imaging of retinal layers. Following a cerebral infarction of the retrogeniculate visual pathway thinning of the retinal nerve fiber layer (RNFL) and ganglion cell layer (GCL) occur as correlates of retrograde transsynaptic neuroaxonal loss (6–13). The only longitudinal study in the relatively early stages post-stroke with 7 patients demonstrated a range of peripapillary RNFL thickness rate reduction from 0.9 to 6.3 μm for every 100 days of elapsed time (17). In the cross-sectional part, a pronounced RNFL degeneration in the first couple of years with a rate of about 9 μm per log year disease duration, which then slowed down in later years, was presented (17). Knowledge of predictors and risk factors for chronic disability is warranted for the development of proactive individualized treatment strategies and could facilitate clinical trials by elucidating the pathophysiology and natural disease course as well as yielding potential surrogate outcome parameters. The objectives of this case series were the detection and characterization of possible transsynaptic retrograde degeneration (TRD) into the retina after PCA infarctions in humans with OCT and multifocal visual evoked potentials (mfVEPs).

## Patients and Methods

### Ethics

The local ethics committee of Heinrich Heine University Duesseldorf [Ethikkomission der Medizinischen Fakultät der Heinrich-Heine-Universität Düsseldorf] approved this prospective observational case series (Number 4436R). Written informed consent was obtained from all participants in accordance with the Declaration of Helsinki.

### Patients

Patients were recruited from our stroke unit at the Department of Neurology, Heinrich-Heine-University Duesseldorf, Germany. Inclusion criteria were subjective visual field defects, age ≥ 18 years, informed consent and a symptomatic ischemic lesion on CT or MRI involving the retrogeniculate visual pathway within two weeks from the onset of clinical symptoms.

Brain lesions compatible with the clinical symptoms and their onset showing hypoattenuation of brain tissue in CT or increased diffusion-weighted imaging (DWI) signal and reduced apparent diffusion coefficient (ADC) values in MRI in the acute phase were judged as cerebral infarcts by our neuroradiology department. All ischemic lesions judged clinically relevant were included. We defined no minimum extension of the ischemic lesion.

Exclusion criteria were severe anomaly of refraction, defined as defined as > 6 dpt, history of surgery of any eye, confounding ophthalmologic diseases as defined by the OSCAR-IB criteria (18), chronic neurodegenerative disorders and old lesions within the visual pathway detectable on MRI or CT imaging (for the list of inclusion and exclusion criteria, see Table S1).

After soliciting written informed consent using the approved informed consent forms, participants underwent multimodal, non-invasive tests (as listed in Table S2) and were followed up at 6 months post stroke in our outpatient clinic.

The ocular exclusion criteria according to the OSCAR-IB criteria (18) were ruled out by a neuro-ophthalmologic examination including funduscopy, impression tonometry, slit lamp examination and assessment of the medical history.

Out of 30 patients with PCA infarcts recruited from our stroke unit at the Heinrich-Heine-University Duesseldorf from 2013 to 2017, 4 were excluded because of systemic or retinal diseases, 5 were excluded because insufficient OCT data/quality.

### Optical Coherence Tomography (OCT)

The study was performed and is reported in accordance with the APOSTEL recommendations (19). Spectral-domain OCT (SD-OCT, Spectralis®, Heidelberg Engineering) was performed to measure the thickness of specific retinal layers namely the RNFL and the inner retinal layers (IRL) consisting of RNFL, ganglion cell layer and inner plexiform layer in macular volume scans (30° × 25°, 13 ART, high speed, 25 B-scans) being our primary retinal outcome parameters. Other OCT parameters included the complex of the ganglion cell layer and inner plexiform layer (GCIPL), inner nuclear layer (INL), outer plexiform layer (OPL), and outer nuclear layer (ONL) in macular cubes. Furthermore, the peripapillary retinal nerve fiber layer (pRNFL) was assessed in high resolution ring scans centered on the optic disc (12°, 100 ART, high resolution). The different peripapillary nasal (N), nasal inferior (NI), nasal superior (NS), temporal (T), temporal inferior (TI), and temporal superior (TS) sectors were analyzed.

The retinal layers including the GCIPL were assessed by semi-automated segmentation using the segmentation algorithm of the eye explorer software (version 1.8.6.0, Heidelberg Engineering) and manual correction of obvious segmentation errors by a blinded rater.

### Multifocal VEPs (mfVEPs)

MfVEPs were performed using the Visionsearch® mfVEP device according to the manufacturer's instructions as described elsewhere (20, 21). In short, monocular stimulation will be performed using the Visionsearch® device applying simultaneous multi-focal stimulation of 56 segments of the visual field (24 degrees of eccentricity) via a 68 second pseudorandom sequence and recording a 2-channel visual response using a custom designed occipital cross electrode holder which predetermines the four occipital electrode positions (20, 21).

### Statistical Evaluation

Statistical analyses were performed using SPSS Statistics 20 (IBM). Kolmogorov Smirnov test was performed on all outcome parameters and revealed normal distribution. A two-tailed paired *t*-test was used to analyze for differences of OCT parameters between the sides ipsilateral and contralateral to the infarction. Furthermore, the longitudinal changes of the OCT parameters were analyzed comparing the differences between baseline and follow-up in a two tailed paired *t*-test. As the number of patients for mfVEP at baseline and follow-up analysis was smaller, Wilcoxon rank sum test was chosen for mfVEP analysis. A significance level of 0.05 was used.

## Results

### Patients

Twenty one patients with acute PCA infarction were included according to our inclusion and exclusion criteria (Table S1). Among these 21 patients 3 patients had bilateral PCA infarctions. Three patients had additional cerebellar infarctions. The delay between symptom onset and CT or MRI imaging was 4.08 ± 3.13 days (mean ± SD). The baseline characteristics of the patients are presented in Table 1.

**Table 1:** Baseline characteristics of the PCA infarction patients.

	***N* = 21**
Median age in years (interquartile range)	64 (54.5–80)
Male gender	11 (52%)
Arterial hypertension	16 (76%)
Diabetes mellitus	3 (14%)
Vascular disease (prior myocardial infarction, peripheral artery disease, or aortic plaque)	4 (19%)
Right PCA infarct location	13 (62%)
Bilateral PCA infarct location	3 (14%)
Stroke etiology	
Cardioembolic	10 (47%)
Cryptogenic	10 (47%)
Macroangiopathic (large-artery atherosclerosis due to > 50% of the basilary artery)	1 (6%)
Mean corrected visual acuity in decimals (±SD)	0.83 (± 0.024)
Mean intraocular pressure in mmHg (±SD)	15.4 (± 2.7)
Mean symptom onset to CT or MRI imaging time in days (±SD)	4.08 (± 3.13)
Mean symptom onset to first OCT assessment in days (±SD)	6.00 (± 3.14)
Mean symptom onset to first mfVEP assessment in days (±SD)	9.36 (± 8.27)

### OCT at Baseline

**Figure 1:**
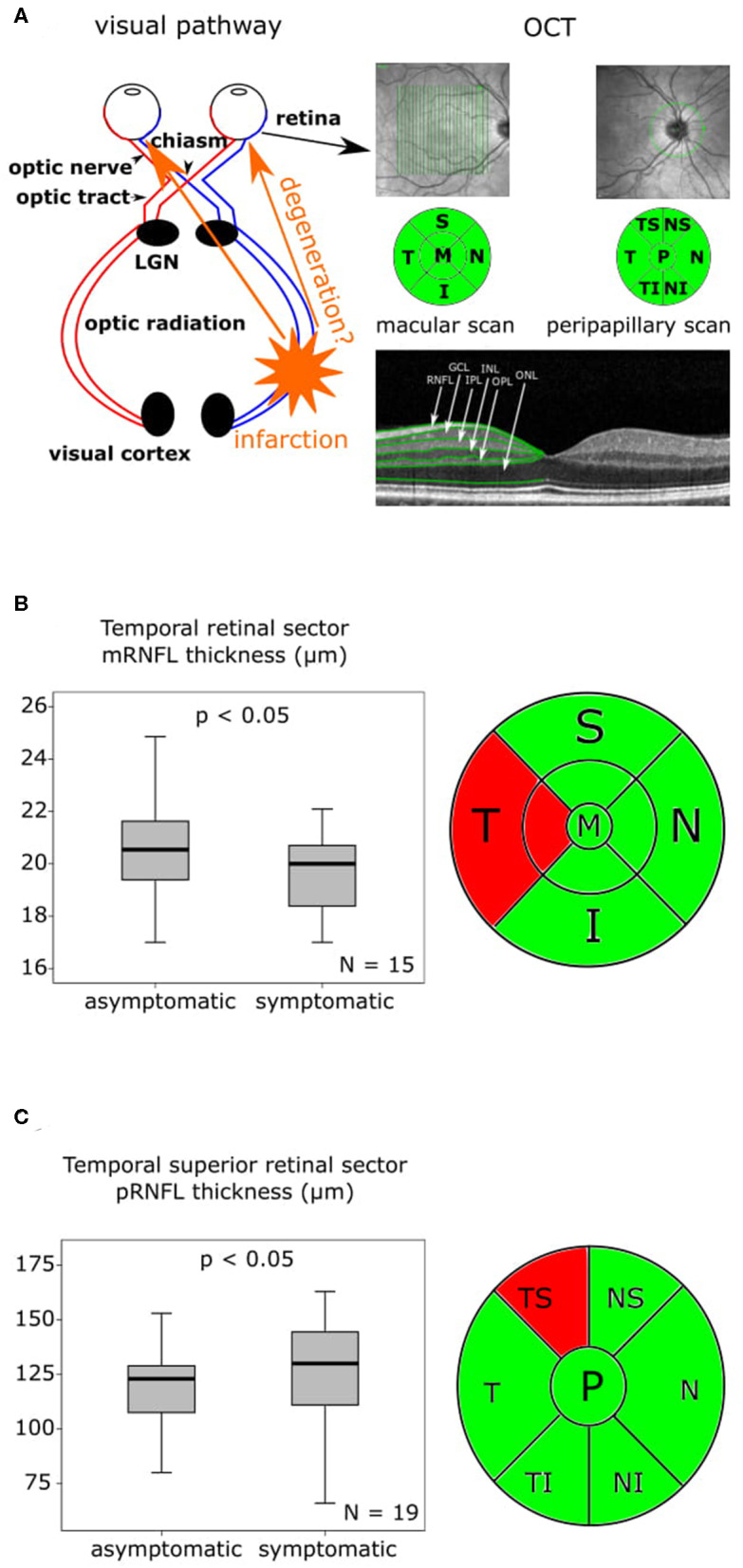
OCT findings at baseline and 6-month follow-up are presented as boxplots. The horizontal lines in the middle of the boxplots demonstrate the medians. The interquartile range (IQR) is presented by the box, and the minimum and maximum values by whiskers (excluding outliers). Outliers defined as values 1.5 to 3.0 times outside the IQR are presented as circles and extreme outliers defined as values of more than 3.0 times outside the IQR are presented as asterisks. **(A)** On the left a schematic representation of the visual pathway with the assumed transsynaptic retrograde degeneration (TRD), on the right an exemplary optical coherence tomography (OCT) fundus image of the macular and peripapillary area with the green sector grids [macular: superior (S), nasal (N), inferior (I), temporal (T) and peripapillary: nasal superior (NS), nasal (N), nasal inferior (NI), temporal inferior (TI), temporal (T), temporal superior (TS)] and a segmentation of an OCT Scan indicating the different layers. **(B)** On the left the mRNFL thickness of the temporal retinal sector at baseline with a significant (*p* < 0.05) asymmetry of the symptomatic side compared to the asymptomatic side, *N* = 15, on the right the macular OCT sector grid with the thinner temporal sector marked in red. **(C)** On the left the pRNFL thickness of the temporal superior retinal sector at baseline with a significant (*p* < 0.05) asymmetry of the symptomatic side compared to the asymptomatic, *N* = 19, on the right the peripapillary OCT sector grid with the affected temporal superior sector marked in red.

Complete OCT scans of both eyes were obtained from all patients and revealed no structural abnormalities in any of the retinal layers or in the pigment epithelium in any of the subjects meeting the inclusion criteria. A schematic representation of the visual pathway with the assumed transsynaptic retrograde degeneration (TRD) and exemplary OCT fundus images of the macular and peripapillary areas are presented in Figure 1A as well as OCT sector grids and an OCT scan with segmented retinal layers. At baseline examination 6.00 ± 3.14 days (mean ± standard deviation (SD)) after the initial stroke event, the findings (Table S3) differed in the temporal and nasal sector of the macular scans (Table S3, Figures S1A,B). The temporal sector already presented a significant difference of the macular RNFL (mRNFL) thickness of the symptomatic side ipsilateral to the infarction compared to the contralateral side (Table S3, Figure 1B), while we observed no differences in mRNFL thickness of the nasal sector (Table S3, Figure S1A). No differences of the macular GCIPL (mGCIPL) and macular IRL (mIRL) were detected (Figures S1C–F). The peripapillary ring scans (Table S3, Figures S1G,H) revealed a significant asymmetry of peripapillary RNFL (pRNFL) thickness (*p* < 0.05) in the symptomatic temporal superior sector compared to the asymptomatic side at baseline (Table S3, Figure 1C).

### OCT Follow-Up

**Figure 2:**
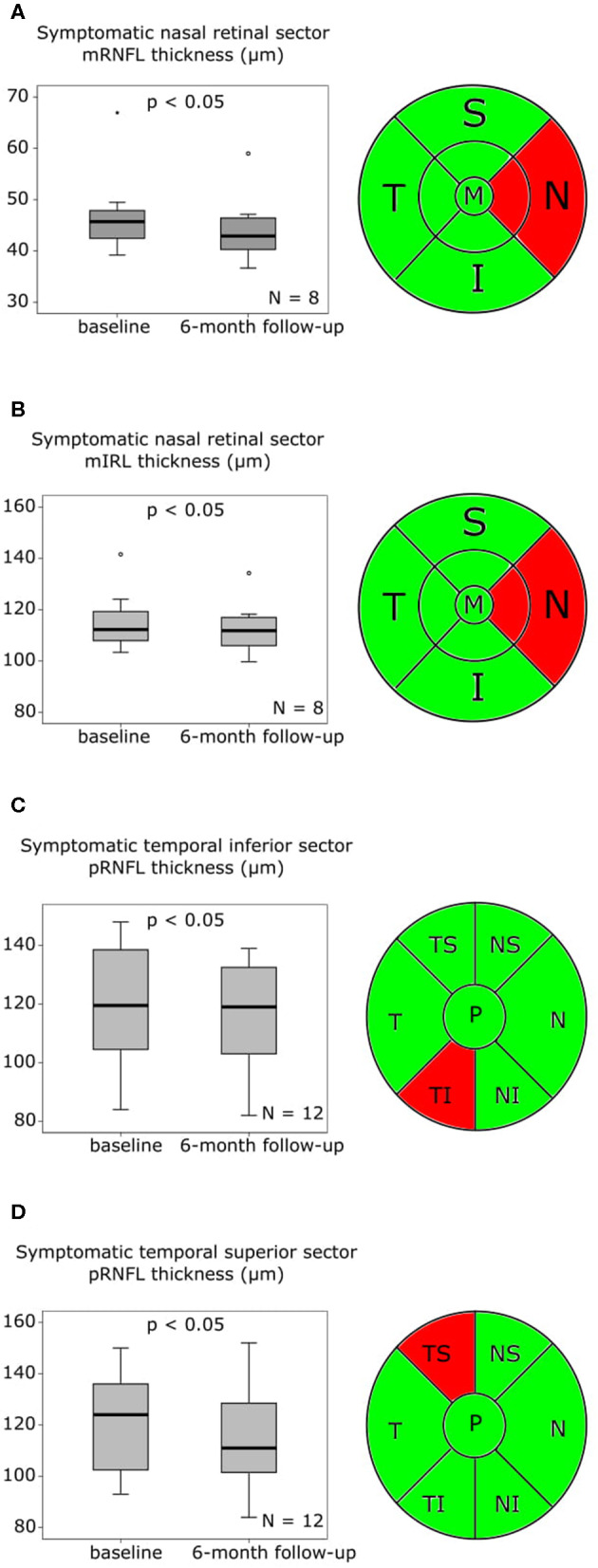
OCT findings baseline to follow-up after 6 months are presented as boxplots. The horizontal lines in the middle of the boxplots demonstrate the medians. The interquartile range (IQR) is presented by the box, and the minimum and maximum values by whiskers (excluding outliers). Outliers defined as values 1.5 to 3.0 times outside the IQR are presented as circles and extreme outliers defined as values of more than 3.0 times outside the IQR are presented as asterisks. **(A)** On the left the macular retinal nerve fiber layer (mRNFL) thickness of the symptomatic nasal retinal sector with a significant (*p* < 0.05) reduction at follow-up after 6 months compared to baseline, *N* = 8, on the right the macular optical coherence tomography (OCT) sector grid with the affected nasal sector marked in red. **(B)** On the left the macular inner retinal layer (mIRL) thickness of the symptomatic nasal retinal sector at baseline with a significant (*p* < 0.05) reduction at follow-up after 6 months compared to baseline, *N* = 8, on the right the macular OCT sector grid with the affected nasal sector marked in red. **(C)** On the left the peripapillary retinal nerve fiber layer (pRNFL) thickness of the symptomatic temporal inferior retinal sector with a significant (*p* < 0.05) reduction at follow-up after 6 months compared to baseline, *N* = 12, on the right the peripapillary OCT sector grid with the affected temporal inferior sector marked in red. **(D)** On the left the pRNFL thickness of the symptomatic temporal superior retinal sector with a significant (*p* < 0.05) reduction at follow-up after 6 months compared to baseline, *N* = 12, on the right the peripapillary OCT sector grid with the affected temporal superior sector marked in red.

At the follow-up, which was performed after a mean of 176 ± 6.82 SD days (6 months) macular OCT scans were available in eight, and peripapillary OCT scans in 12 patients. In our eight patients, who were available for macular OCT follow-up assessments (Table S4, Figures S2A-F), the mRNFL thickness (Table S4, Figure 2A) and the mIRL thickness (Table S4, Figure 2B) of the symptomatic nasal retinal sector was significantly reduced compared to baseline. The symptomatic temporal sector did not show further longitudinal changes beyond the differences observed at baseline. In the follow-up analysis of the peripapillary OCT scans after 6 months (Table S4, Figures S2G–J), the symptomatic pRNFL in 12 available patients was significantly reduced compared to baseline in the temporal inferior (*p* < 0.05) and temporal superior (*p* < 0.05) sector (Table S4, Figures 2C,D).

### mfVEP at Baseline

**Figure 3:**
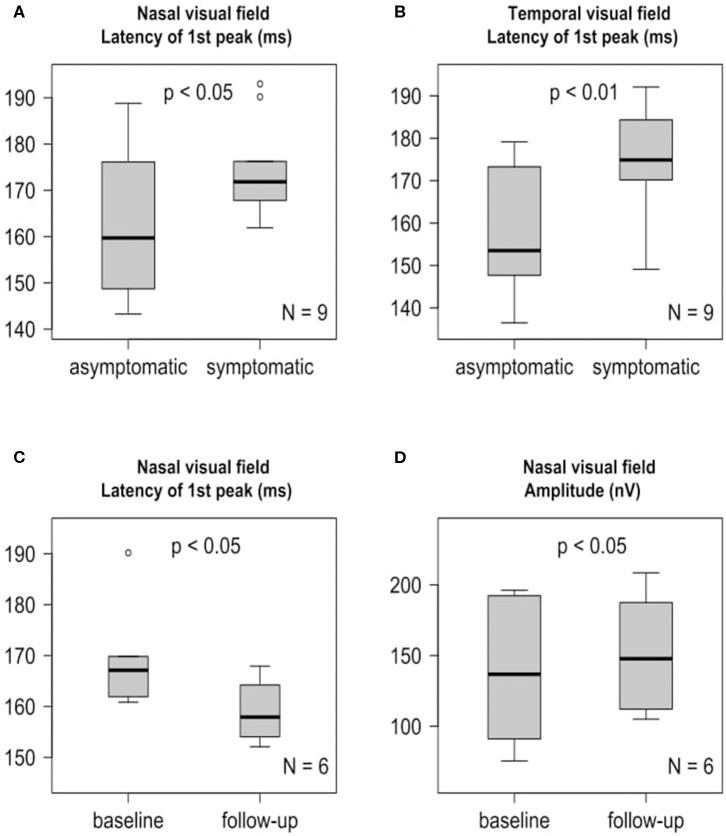
mfVEP findings at baseline and 6-month follow-up are presented as boxplots. The horizontal lines in the middle of the boxplots demonstrate the medians. The interquartile range (IQR) is presented by the box, and the minimum and maximum values by whiskers (excluding outliers). Outliers defined as values 1.5 to 3.0 times outside the IQR are presented as circles and extreme outliers defined as values of more than 3.0 times outside the IQR are presented as asterisks. **(A)** Significant (*p* < 0.05) first peak latency difference of the symptomatic nasal visual field and the asymptomatic nasal visual field at baseline, *N* = 9. **(B)** Significant (*p* < 0.01) first peak latency difference of the symptomatic temporal visual field and the asymptomatic temporal visual field at baseline, *N* = 9. **(C)** Significant (*p* < 0.05) earlier symptomatic nasal visual field first peak latency after 6 months compared to baseline, *N* = 6. **(D)** Significant (*p* < 0.05) increase of the symptomatic nasal visual field amplitude after 6 months compared to baseline, *N* = 6.

The baseline examination was performed 9.36 ± 8.27 days (mean ± SD) after the initial stroke event. Analyzing the 9 available baseline mfVEPs (Table S5, Figures S3A–D), we observed a significant delay in first peak latency of the symptomatic affected nasal (Table S5, Figure 3A) and temporal (Table S5, Figure 3B) visual fields contralateral to the infarction compared to the asymptomatic sides. The amplitudes did not differ significantly between the symptomatic and asymptomatic side, neither for the nasal (Table S5, Figure S3C) nor for the temporal sector (Table S5, Figure S3D).

### mfVEP Follow-Up

Longitudinal follow up mfVEPs (Table S6, Figures S3E–H) were available for 6 patients. At the follow up, which was performed 6 months after stroke, we observed a partial recovery of the first peak latency (Table S6, Figure 3C) and amplitude (Table S6, Figure 3D) of the symptomatic nasal visual fields contralateral to the infarction compared to baseline. Interestingly, this was not the case for the temporal visual fields (Table S6, Figures S3F,H).

## Discussion

In our case series, we observed a significant−2.92 ± 2.53 μm (mean ± SD) reduction of the mRNFL, a−3.75 ± 5.46 μm (mean ± SD) reduction of the temporal inferior pRNFL sector, a−5.75 ± 8.01 μm (mean ± SD) reduction of the temporal superior pRNFL sector and a −2.56 ± 2.94 μm (mean ± SD) mIRL reduction in the symptomatic nasal retinal sector after 6 months compared to baseline. Furthermore, at baseline the symptomatic pRNFL showed an elevated temporal superior sector compared to the asymptomatic side, while after 6 months we observed a reduction of the symptomatic pRNFL in the temporal inferior and temporal superior sectors. Our mfVEP results support the finding that the dynamics of degeneration and regeneration might differ between the nasal and temporal retinal sector. The symptomatic nasal visual fields showed significant signs of partial recovery after 6 months in amplitude and first peak latency compared to baseline, while the symptomatic temporal visual fields did not. Our OCT results are in line with the previously reported pronounced pRNFL degeneration of 9 μm per log year disease duration in the first couple of years and a range of peripapillary RNFL thickness rate reduction from 0.9 to 6.3 μm for every 100 days of elapsed time (17). The finding of an elevated pRNFL thickness of the temporal superior sector at baseline may suggest an early swelling as a result of the lesion in the posterior visual pathway. The reason for this swelling remains subject to speculation but could involve edema due to inflammatory processes like microglial activation resulting from very early TRD. The peripapillary and macular results are not contradictory as the temporal peripapillary fibers are in the area of the macular nasal sector, which in contrast to the temporal sector showed no volume reduction at baseline. The predominance of degeneration of the affected temporal sector on the ipsilateral side of the pRNFL over time is consistent with a previous report (22). The reduction of the macular IRL is in accordance with previous studies reporting macular ganglion cell complex thinning of the corresponding symptomatic hemifields or sectors after PCA infarctions (8, 22). Interestingly, we detected a very early reduction of the macular RNFL in the symptomatic temporal retinal sector already at baseline although the lateral geniculate nucleus (LGN) or the optic tract were not affected on MR or CT imaging in our patients. A previous study reported a detectable peripapillary RNFL thinning after 100 days of the insult with greater loss occurring in the first years with a rate of about 9μm per log year (17). Possibly the macular RNFL is more sensitive for change than the peripapillary measure. Previous studies on other disease entities have reported macular RNFL thinning in the absence of pRNFL changes (23, 24). Naito et al. (25) have demonstrated that the projections of crossing fibers from the nasal region of the retina have a larger sector on the nasal than on the temporal side of the optic disc. It is discussed that there is considerably more intermingling of crossed and uncrossed fibers on the temporal quadrant (25), which may partly explain our results of a faster degeneration of the temporal retinal sector and a better recovery of the corresponding nasal visual field after 6 months. TRD of retinal ganglion cells and their axons after occipital lobe damage involving the visual pathway is discussed to result from a loss of the postsynaptic target (6, 8, 9, 17, 26). The effects of occipital damage of the visual pathway have been investigated in animal models. After removal of all or parts of the striate cortex in macaque monkeys, the corresponding region of the dorsal lateral geniculate nucleus incurs an almost complete loss of projection neurons within 12 weeks (27). TRD of retinal ganglion cells and atrophy of the lateral geniculate nucleus have been shown 48 months after occipital cortex ablation in monkeys (28, 29). Electrophysiological examinations using pattern-evoked electroretinogram studies in the 1990s detected differences in signal strengths between the two hemiretinae of patients suffering from homonymous hemianopia as signs of TRD (30, 31). Case series and small cross-sectional investigations have reported a reduction of the RNFLand macular GCL following cerebral infarction providing evidence of transsynaptic retrograde retinal ganglion cell degeneration (6–13). In the cross-sectional part of a study with occipital lobe damage a pronounced peripapillary RNFL degeneration was detected in the first couple of years with a rate of about 9 μm per log year disease duration, which then slowed down in later years (17). In the same study the only small longitudinal investigation of the relatively early stages post-stroke in 7 patients with hemianopia demonstrated a range of the peripapillary RNFL thickness rate reduction from 0.9 to 6.3 μm for every 100 days of elapsed time (17). A strength of our case series was that not only structure (OCT) but also function (mfVEP) were quantitatively investigated. In addition, longitudinal design allowed us to analyze the OCT and mfVEP parameters over time. To our knowledge, we present the first case series using OCT and mfVEP to investigate the changes after PCA infarctions cross-sectionally and longitudinally. Despite the reportedly good test-retest reliability of OCT und mfVEP (32–34) we cannot rule out sampling artifacts resulting from the relatively small sample size of our study. Another limitation is the inclusion of patients with different extensions of PCA infarctions identified by MRI or CT scans, which additionally might miss smaller lesions in other brain areas, especially in the case of CT. We therefore cannot completely rule out concomitant additional direct ischemic insults to the optic tract and LGN (35, 36) beyond the infarctions identified. Such infarctions could have caused the observed retinal layer changes by direct retrograde axonal degeneration, rather than by TRD. However, one would expect that these lesions would have occurred in a random distribution on both the ipsilateral and contralateral side of the PCA infarction rather than only on the ipsilateral side where we observed the degeneration in our study. In addition, patients with ischemic stroke may have a considerable risk of concomitant ischemic damage to the anterior visual pathways based on their vascular risk factors (36). However, as stated above, this would affect the anterior visual pathway of both eyes. Another limitations of our study are that no control subjects with reference OCT and mfVEP data were included and that besides confrontation visual testing with finger perimetry formal perimetric investigations of visual fields and/or electroretinograms as direct functional retinal readouts were not available. In conclusion, our case series support previous reports that TRD occurs early after PCA infarctions involving the visual system. The differential dynamics of degeneration and functional recovery of the nasal and temporal parts of the retina give new insights into the time course of TRD. This observation warrants further functional mechanistic investigations including complementary standardized MRI studies with tractography and lesion mapping, visual field testing, and electroretinograms as well as confirmation in larger cohorts. These findings may contribute to a better understanding of TRD, which might have implications for future treatment options and recovery strategies in different time windows.

## Data Availability Statement

The datasets generated for this study are available on request to the corresponding author.

## Ethics Statement

The studies involving human participants were reviewed and approved by Ethikkomission der Medizinischen Fakultät der Heinrich-Heine-Universität Düsseldorf. The patients/participants provided their written informed consent to participate in this study.

## Author Contributions

J-IL contributed to the study design, data acquisition, data analysis, drafting of the manuscript, revision of the manuscript for important intellectual content. LB contributed to the data acquisition, data analysis, drafting of the manuscript, revision of the manuscript for important intellectual content. LG and RG contributed to the data acquisition, revision of the manuscript for important intellectual content. JH contributed to the data analysis and revision of the manuscript for important intellectual content. OA, MG, SJ, and H-PH contributed to the revision of the manuscript for important intellectual content. PA contributed to the study design, data acquisition, data analysis, drafting of the manuscript, revision of the manuscript for important intellectual content.

## Conflict of Interest

J-IL has received honoraria for speaking/consultation from Bayer Healthcare, Boehringer Ingelheim, Novartis, Allergan, Ipsen, Teva Pharmaceuticals and Daiichi-Sankyo as well as travel grants from Bayer Healthcare, Merz Pharmaceuticals, Ipsen and Allergan outside the submitted work. OA has received honoraria for speaking/consultation and travel grants from Bayer Healthcare, Biogen Idec, Chugai, Novartis, Medimmune, Merck Serono, and Teva and research grants from Bayer Healthcare, Biogen Idec, Novartis, and Teva, outside the submitted work. MG has received honoraria for speaking/consultation from Bayer Healthcare, Boehringer Ingelheim and a research grant from B. Braun, outside the submitted work. SJ has received honoraria for speaking/consultation from Bayer Healthcare, Boehringer Ingelheim, Bristol-Myers Squibb, Pfizer, Biogen, Alexion and Daiichi-Sankyo as well as travel grants from Bayer Healthcare and Daiichi-Sankyo, outside the submitted work. H-PH has outside the work presented here received fees for serving on steering or data monitoring commitees from Bayer Healthcare, Biogen, Celgene Receptos, GeNeuro, Sanofi Genzyme, Merck, Novartis, Octapharma, Teva Pharmaceuticals, MedImmune, and Roche, fees for serving on advisory boards from Biogen Idec, Sanofi Genzyme, Merck, Novartis Pharmaceuticals, Octapharma, Teva Pharmaceuticals, and Roche, and lecture fees from Biogen, Sanofi Genzyme, Merck, Novartis Pharmaceuticals, Octapharma, Teva Pharmaceuticals, MedImmune, and Roche. PA reports grants, personal fees and non-financial support from Allergan, Biogen, Ipsen, Merz Pharmaceuticals, Novartis, and Roche, personal fees and non-financial support from Bayer Healthcare, Merck, and Sanofi-Aventis/Genzyme, outside the submitted work. The remaining authors declare that the research was conducted in the absence of any commercial or financial relationships that could be construed as a potential conflict of interest.

## Abbreviations

TRD: transsynaptic retrograde degeneration
PCA: posterior cerebral artery
OCT: optical coherence tomography
SD-OCT: spectral-domain optical coherence tomography
mfVEPs: multifocal visual evoked potentials
SD: standard deviation
RNFL: retinal nerve fiber layer
mRNFL: macular retinal nerve fiber layer
pRNFL: peripapillary retinal nerve fiber layer
VFDs: visual field defects
GCL: ganglion cell layer
ADC: apparent diffusion coefficient
DWI: diffusion-weighted imaging
MRI: magnetic resonance imaging
CT: computed tomography
dpt: diopters
IRL: inner retinal layer
GCIPL: ganglion cell layer and inner plexiform layer
INL: inner nuclear layer
OPL: outer plexiform layer
ONL: outer nuclear layer
N: nasal
NI: nasal inferior
NS: nasal superior
T: temporal
TI: temporal inferior
TS: temporal superior
LGN: lateral geniculate nucleus.
