# Supplementary material for: Retinal Changes After Posterior Cerebral Artery Infarctions Display Different Patterns of the Nasal und Temporal Sector in a Case Series
Source: Front Neurol. 2020 Jun 5;11:508. doi: 10.3389/fneur.2020.00508 (PMC7290045; doi:10.3389/fneur.2020.00508)
Supplement: Supplementary file 1 [file Data_Sheet_1.pdf]

Supplementary information

Table S1: Inclusion and exclusion criteria

**Inclusion criteria**	**Exclusion criteria**
• Subjective visual field defects<br>• Age ≥ 18 years<br>• Informed consent of a legally competent subject<br>• Ischemic lesion on CT or MRI involving the retrogeniculate visual pathway with clinical symptoms within the last two weeks	• Severe anomaly of refraction, defined as > 6 dpt<br>• Operation of an eye in the medical history<br>• Confounding ophthalmologic diseases as defined by OSCAR-IB criteria (18)<br>• Chronic neurodegenerative disorders<br>• Old lesions within the visual pathway detectable on MRI or CT imaging

Table S2: Examinations during recruitment, baseline and follow-up

**Recruitment and inclusion/exclusion criteria**<br>CT or MRI with ischemic lesion involving the retrogeniculate visual pathway<br>Ophthalmological examinations<br>Visual acuity testing<br>Patient characteristics
**Baseline**<br>SD-OCT<br>mfVEPs<br>Patient characteristics
**Follow-up**<br>SD-OCT<br>mfVEPs<br>Patient characteristics

Table S3: OCT findings at baseline comparing asymptomatic to symptomatic sectors, the analysis considers all available patients: Means, mean differences (mean symptomatic minus mean asymptomatic), standard deviations and p-values are provided for the thickness of the nasal and temporal sectors of the macular retinal layers (n=15) mRNFL, mGCIPL, mIRL and the six sectors (nasal, nasal inferior, nasal superior, temporal, temporal inferior, temporal superior) of the peripapillary RNFL (pRNFL) (n=19) at baseline, comparing the symptomatic and the asymptomatic side, n.s. indicating no significant difference. P-values <0.05 were considered as statistically significant (two-tailed paired t-test).

**OCT**	**Mean asymptomatic**	**+/- SD**	**Mean symptomatic**	**+/- SD**	**Mean difference (mean symptomatic**	**+/- SD**	**p-value**

					**minus mean asymptomatic)**		
**mRNFL (µm) nasal, n=15**	46.98	8.01	46.67	6.92	-0.31	6.57	n.s.
**mRNFL (µm) temporal , n=15**	20.67	1.96	19.61	1.58	-1.05	1.65	**<0.0 5**
**mGCIPL (µm) nasal, n=14**	68.94	8.64	68.35	9.09	-0.59	3.81	n.s.
**mGCIPL (µm) temporal , n=14**	70.78	7.37	69.42	9.74	-1.36	5.10	n.s.
**mIRL (µm) nasal, n=15**	115.67	14.5 4	114.80	14.1 7	-0.87	5.45	n.s
**mIRL (µm) temporal , n=15**	91.32	7.70	89.28	9.13	-2.04	3.90	n.s.
**pRNFL (µm) nasal; n=19**	59.74	11.3 0	61.05	9.05	1.32	9.91	n.s.
**pRNFL (µm) nasal inferior, n=19**	103.95	25.5 0	106.37	18.0 7	2.42	16.1 1	n.s.
**pRNFL (µm) nasal superior, n=19**	98.16	22.4 2	108.37	20.0 9	10.21	24.5 2	n.s.
**pRNFL (µm) temporal , n=19**	77.16	10.8 0	77.68	8.65	0.53	7.76	n.s.
**pRNFL (µm) temporal inferior, n=19**	120.74	21.1 6	118.53	24.4 0	-2.21	16.8 2	n.s.

**pRNFL (µm) temporal superior, n=19**	119.11	19.91	126.79	24.92	7.68	14.75	**<0.05**

Table S4: OCT findings comparing symptomatic nasal and temporal sectors at baseline to findings after 6 months, the analysis considers all available patients: Means, mean differences (6 months minus baseline), standard deviations and p-values are provided for the thickness of the symptomatic nasal and temporal sectors of the macular retinal layers mRNFL, mGCIPL, mIRL and the symptomatic six sectors (nasal, nasal inferior, nasal superior, temporal, temporal inferior, temporal superior) of the peripapillary RNFL (pRNFL) comparing baseline and 6 months follow-up findings, n.s. indicating no significant difference. P-values <0.05 were considered as statistically significant (two-tailed paired t-test).

OCT	Mean baseline	+/-SD	Mean after 6 months	+/-SD	Mean difference (6 months minus baseline)	+/-SD	p-value
**mRNFL (µm) nasal symptomatic, n=8**	47.28	8.53	44.36	6.84	-2.92	2.53	**<0.05**
**mRNFL (µm) temporal symptomatic, n=10**	19.35	1.69	19.19	1.77	-0.16	0.62	n.s.
**mGCIPL (µm) nasal symptomatic, n=7**	68.65	5.98	68.97	6.35	0.32	2.34	n.s.
**mGCIPL (µm) temporal symptomtic, n=9**	68.91	6.98	71.06	12.04	2.15	10.01	n.s.
**mIRL (µm) nasal symptomatic, n=8**	115.48	12.27	112.93	10.57	-2.56	2.94	**<0.05**
**mIRL (µm) temporal symptomatic, n=10**	88.22	7.25	87.57	7.89	-0.64	1.95	n.s.
**pRNFL (µm) nasal**	58.91	8.18	57.55	7.17	-1.36	4.13	n.s.

**symptomatic, n=11**							
**pRNFL (µm) nasal inferior symptomatic, n=11**	103.73	20.68	108.91	27.54	5.18	23.64	n.s.
**pRNFL (µm) nasal superior symptomatic, n=11**	102.27	19.78	100.64	19.49	-1.64	12.31	n.s.
**pRNFL (µm) temporal symptomatic, n=12**	74.83	8.34	73.92	8.92	-0.92	3.15	n.s.
**pRNFL (µm) temporal inferior symptomatic, n=12**	120.25	20.25	116.50	18.64	-3.75	5.46	**<0.05**
**pRNFL (µm) temporal superior symptomatic, n=12**	120.75	18.56	115.00	19.18	-5.75	8.01	**<0.05**

Table S5: mfVEP findings at baseline comparing asymptomatic to symptomatic sectors, the analysis considers all available patients: Means, mean differences (mean symptomatic minus mean asymptomatic), standard deviations and p-values are provided for the amplitude and first peak latency of the nasal and temporal sectors at baseline, comparing the symptomatic and the asymptomatic side, n.s. indicating no significant difference. P-values <0.05 were considered as statistically significant (Wilcoxon rank sum test).

**mfVEP baseline**	**Mean asymptomatic**	**+/-SD**	**Mean symptomatic**	**+/-SD**	**Difference (symptomatic minus asymptomatic)**	**+/-SD**	**p-value**
**Amp (nV) nasal, n=9**	148.63	32.813	135.50	45.763	-9.0978	51.19113	n.s.
**Amp (nV) temporal, n=9**	127.16	73.930	131.33	40.473	4.1636	88.26740	n.s.
**1st peak latency (ms) nasal, n=9**	162.81	17.188	172.53	10.558	11.6933	11.16884	**<0.05**
**1st peak latency (ms) temporal, n=9**	157.53	15.553	172.24	13.259	16.6256	7.97112	**<0.01**

Table S6: mfVEP findings comparing baseline symptomatic nasal and temporal sectors to follow-up findings after 6 months, the analysis considers all available patients: Means, mean differences (6 months minus baseline), standard deviations and p-values are provided for the amplitude and first peak latency of the symptomatic nasal and temporal sectors comparing baseline and 6 months follow up findings, n.s. indicating no significant difference. P-values <0.05 were considered as statistically significant (Wilcoxon rank sum test).

**mfVEP baseline vs follow up**	**Mean baseline**	**+/-SD**	**Mean follow up**	**+/-SD**	**Difference (follow up minus baseline)**	**+/-SD**	**p-value**
**Amp (nV) nasal symptomatic, n=6**	138.1	50.11	151.44	40.85	13.35	13.34	**<0.05**
**Amp (nV) temporal symptomatic, n=6**	138.76	40.07	133.01	33.26	-5.75	34.4	n.s
**1st peak latency (ms) nasal symptomatic, n=6**	169.5	10.70	159.02	6.28	-10.48	8.92	**<0.05**
**1st peak latency (ms) temporal symptomatic, n=6**	169.83	13.92	161.69	10.04	-8.14	10.44	n.s

Supplementary figure legends:

Figure S1: OCT findings at baseline and 6-month follow-up are presented as boxplots. The horizontal lines in the middle of the boxplots demonstrate the medians. The interquartile range (IQR) is presented by the box, and the minimum and maximum values by whiskers (excluding outliers). Outliers defined as values 1.5 to 3.0 times outside the IQR are presented as circles and extreme outliers defined as values of more than 3.0 times outside the IQR are presented as asterisks.
A. macular retinal nerve fiber layer (mRNFL) thickness of the nasal retinal sector at baseline with no significant difference between the asymptomatic und symptomatic side, N=15.
B. mRNFL thickness of the temporal retinal sector at baseline with a significant (p<0.05) asymmetry of the symptomatic side compared to the asymptomatic side, N=15.
C. macular ganglion cell-inner plexiform layer (mGCIPL) thickness of the nasal retinal sector at baseline with no significant difference between the asymptomatic und symptomatic side, N=14.
D. mGCIPL thickness of the temporal retinal sector at baseline with no significant difference between the asymptomatic und symptomatic side, N=14.

E. macular inner retinal layer (mIRL) thickness of the nasal retinal sector at baseline with no significant difference between the asymptomatic und symptomatic side, N=15.
F. mIRL thickness of the temporal retinal sector at baseline with no significant difference between the asymptomatic und symptomatic side, N=15.
G. peripapillary retinal nerve fiber layer (pRNFL) thickness of the nasal superior retinal sector at baseline with no significant difference between the asymptomatic und symptomatic side, N=19
H. pRNFL thickness of the temporal superior retinal sector at baseline with a significant ($p<0.05$) asymmetry of the symptomatic (N=19) side compared to the asymptomatic (N=19) side.

Figure S2: OCT findings baseline to follow-up after 6 months are presented as boxplots. The horizontal lines in the middle of the boxplots demonstrate the medians. The interquartile range (IQR) is presented by the box, and the minimum and maximum values by whiskers (excluding outliers). Outliers defined as values 1.5 to 3.0 times outside the IQR are presented as circles and extreme outliers defined as values of more than 3.0 times outside the IQR are presented as asterisks.
A. macular retinal nerve fiber layer (mRNFL) thickness of the symptomatic nasal retinal sector with a significant ($p<0.05$) reduction at follow-up after 6 months compared to baseline, N=8.
B. mRNFL thickness of the symptomatic temporal retinal sector at baseline and follow-up after 6 months with no significant difference, N=10.
C. macular ganglion cell-inner plexiform layer (mGCIPL) thickness of the symptomatic nasal retinal sector at baseline and follow-up after 6 months with no significant difference, N=7.
D. mGCIPL thickness of the symptomatic temporal retinal sector at baseline and follow-up after 6 months with no significant difference, N=9.
E. macular inner retinal layer (mIRL) thickness of the symptomatic nasal retinal sector at baseline with a significant ($p<0.05$) reduction at follow-up after 6 months compared to baseline, N=8.
F. mIRL thickness of the symptomatic temporal retinal sector at baseline and follow-up after 6 months with no significant difference, N=10.
G. peripapillary retinal nerve fiber layer (pRNFL) thickness of the symptomatic nasal inferior retinal sector at baseline and follow-up after 6 months with no significant difference, N=11
H. pRNFL thickness of the symptomatic temporal inferior retinal sector with a significant ($p<0.05$) reduction at follow-up after 6 months compared to baseline, N=12.
I. pRNFL thickness of the symptomatic nasal superior retinal sector at baseline and follow-up after 6 months with no significant difference, N=11
J. pRNFL thickness of the symptomatic temporal superior retinal sector with a significant ($p<0.05$) reduction at follow-up after 6 months compared to baseline, N=12.

Figure S3: mfVEP findings at baseline and 6-month follow-up are presented as boxplots. The horizontal lines in the middle of the boxplots demonstrate the medians. The interquartile range (IQR) is presented by the box, and the minimum and maximum values by whiskers (excluding outliers). Outliers defined as values 1.5 to 3.0 times outside the IQR are presented as circles and extreme outliers defined as values of more than 3.0 times outside the IQR are presented as asterisks.
A. Significant ($p<0.05$) first peak latency difference of the symptomatic nasal visual field and the asymptomatic nasal visual field at baseline, N=9.
B. Significant ($p<0.01$) first peak latency difference of the symptomatic temporal visual field and the asymptomatic temporal visual field at baseline, N=9.
C. Amplitude of the nasal visual field at baseline with no significant difference between the symptomatic and asymptomatic side, N=9.

D. Amplitude of the temporal visual field at baseline with no significant difference between the symptomatic and asymptomatic side, N=9.
E. Significant ($p<0.05$) earlier symptomatic nasal visual field first peak latency after 6 months compared to baseline, N=6.
F. First peak latency of the temporal visual field after 6 months compared to baseline with no significant difference, N=6.
G. Significant ($p<0.05$) increase of the symptomatic nasal visual field amplitude after 6 months compared to baseline, N=6.
H. Amplitude of the temporal visual field after 6 months compared to baseline with no significant difference, N=6.
