# Supplementary figures and images for: Retinal Changes After Posterior Cerebral Artery Infarctions Display Different Patterns of the Nasal und Temporal Sector in a Case Series
Source: Front Neurol. 2020 Jun 5;11:508. doi: 10.3389/fneur.2020.00508 (PMC7290045; doi:10.3389/fneur.2020.00508)

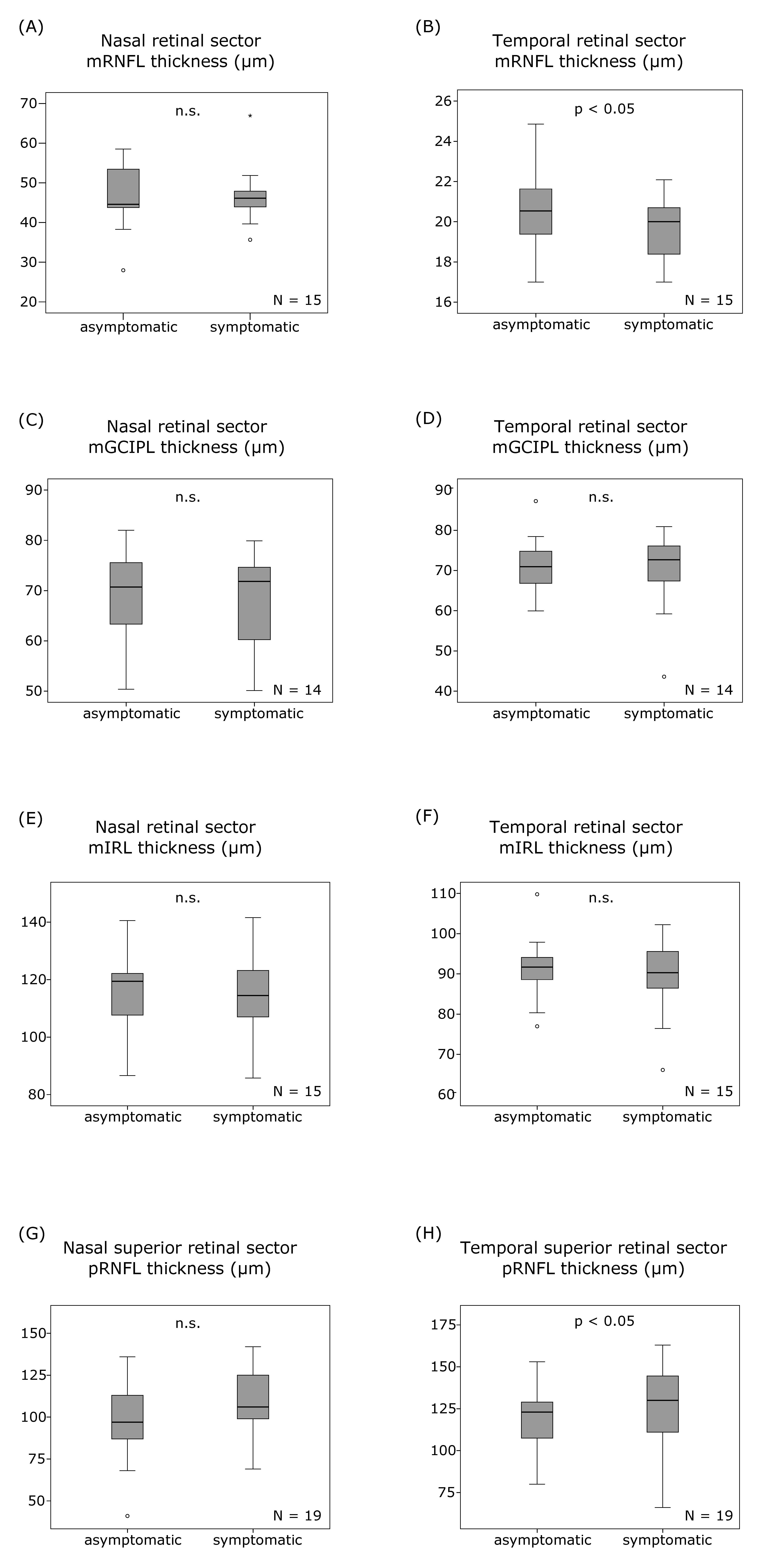

Supplement: Supplementary file 2 [file Image_1.JPEG]

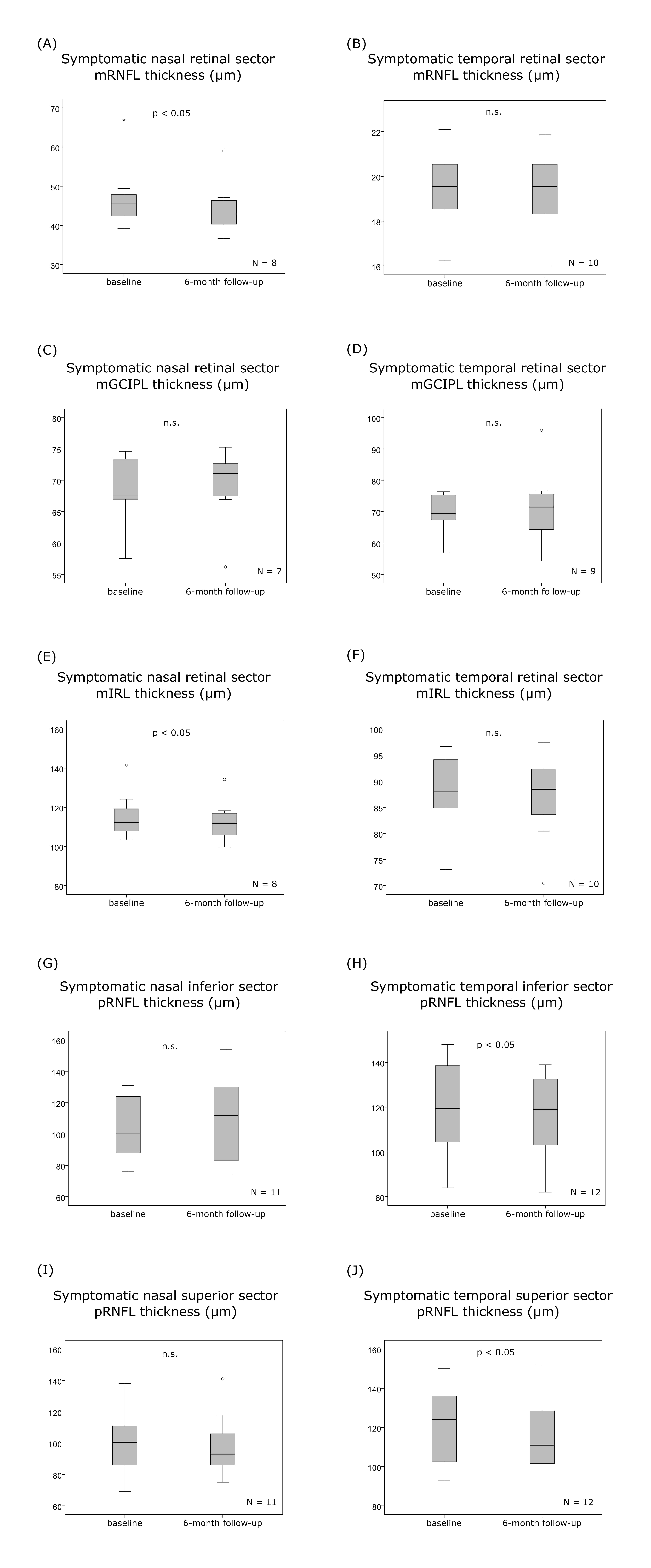

Supplement: Supplementary file 3 [file Image_2.JPEG]

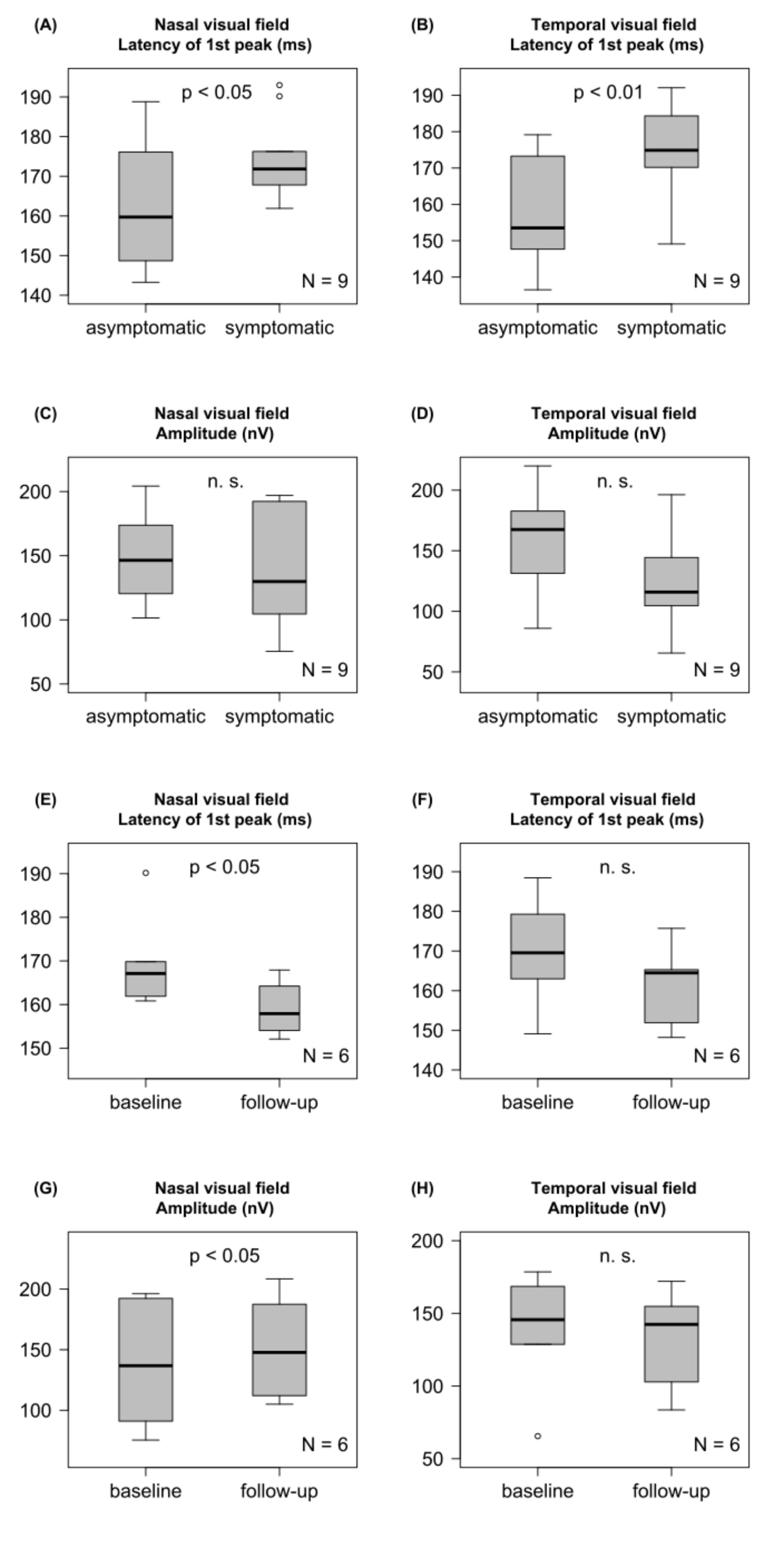

Supplement: Supplementary file 4 [file Image_3.JPEG]
